# A Mindfulness-Based Decentering Technique Increases the Cognitive Accessibility of Health and Weight Loss Related Goals

**DOI:** 10.3389/fpsyg.2018.00587

**Published:** 2018-04-24

**Authors:** Katy Tapper, Zoyah Ahmed

**Affiliations:** Department of Psychology, School of Social Sciences, City, University of London, London, United Kingdom

**Keywords:** mindfulness, decentering, food, weight loss, dieting, goals, health, chocolate

## Abstract

Previous research has shown that a mindfulness-based decentering technique can help individuals resist eating chocolate over a 5-day period. However, it is unclear how this technique exerts its effect. This study explored one potential mechanism; that decentering increases the cognitive accessibility of relevant goals. Male and female participants (*n* = 90) spent 5 min practicing either a decentering or relaxation (control) technique. They then viewed a picture of a chocolate bar for 3 min whilst either applying the decentering technique or letting their mind wander (control). Finally, all participants completed 20 letter strings, rated their motivation for weight loss and for healthy eating, and indicated whether or not they were dieting to lose weight. As predicted, those who had applied the decentering technique produced a greater number of health and weight loss related words when completing the letter strings, compared to those who had simply let their mind wander (*p* < 0.001). However, contrary to predictions, these effects were not significantly greater amongst those who were more motivated to lose weight or eat healthily, or amongst those who were dieting to lose weight, though the means were in the predicted directions. The results suggest that this particular mindfulness technique may increase the accessibility of relevant goals. Further research would be needed to (a) compare effects with other strategies that prompt individuals to remember their goals, (b) examine other potential mechanisms of action, and (c) confirm that effects on self-control are mediated by increased goal accessibility.

## Introduction

Mindfulness-based interventions are increasingly being used to try to alter eating behaviors, reducing food cravings and promote weight management (Katterman et al., 2014; O’Reilly et al., 2014; Mantzios and Wilson, 2015; Olson and Emery, 2015). However, these interventions tend to incorporate a range of different mindfulness- and non mindfulness-based elements. Whilst complex interventions with multiple components may ultimately be more effective than single component interventions (National Institute for Health and Clinical Excellence, 2014), they nevertheless make it difficult to establish the efficacy of individual elements, and to identify underlying mechanisms of action (Tapper, 2017). These issues are important; to develop effective interventions, and to successfully adapt them for different populations and different behaviors, we need to understand how they work. Thus there is a need for more research examining both the effects of specific mindfulness-based strategies (and combinations of strategies) and their mechanisms of action. The current study examines one potential mechanism of action associated with a specific mindfulness-based decentering strategy that has previously been found to influence behavior (Jenkins and Tapper, 2014).

Decentering refers to seeing thoughts and feelings as temporary events that are separate from oneself and not necessarily a true reflection of reality. Decentering has been found to influence food choice (Papies et al., 2015) and reduce chocolate consumption (Moffitt et al., 2012; Jenkins and Tapper, 2014; though other studies have failed to find effects, see Tapper, 2017). Fewer studies have examined potential mechanisms of action.

One possibility is that decentering exerts a bottom–up influence on behavior by dampening affective reactions to stimuli. For example, the grounded theory of cognition states that when a person encounters an object in their environment, they draw on previous experience to simulate interacting with that object, which in turn stimulates similar areas of the brain to real interactions, thus triggering associated bodily responses and feelings of desire (Barsalou, 2008). According to this theory, decentering may bring about immediate reductions in desire by reducing the believability of these mental simulations (Papies et al., 2015). Similarly, the elaborated intrusion theory of desire states that craving arises when transitory thoughts about a particular object are elaborated in working memory (Kavanagh et al., 2005; May et al., 2012, 2015). According to this theory decentering may reduce desire by loading working memory since this will prevent cognitive elaboration that will in turn prevent or interrupt craving development (Tapper, 2018). There is some evidence to suggest that decentering may reduce cravings (Lacille et al., 2014; Schumacher et al., 2017; though see Tapper, 2018 for studies that have failed to find effects).

Alternatively, or additionally, decentering may exert a top–down influence on behavior, improving the individual’s ability to exercise self-control. This may occur through a combination of mechanisms. First, there is evidence to suggest that snacking on high calorie foods may often be habitual (e.g., Adriaanse et al., 2011; Neal et al., 2011; Verhoeven et al., 2012; Cleobury and Tapper, 2014). In other words, snacking may occur, not as a result of a conscious decision, but instead as an automatic response to specific cues. In some instances these cues may be internal events such as thoughts or feelings (Adriaanse et al., 2009). Thus asking a person to decenter from these thoughts and feelings may interrupt the automatic response, allowing them to engage in more controlled decision-making. Second, once the individual is engaging in more controlled decision-making, they may be more likely to think about relevant goals. This may occur where the individual has a history of considering relevant goals when engaging in decision-making in the presence of particular stimuli (such as tempting foods).

Jenkins and Tapper (2014) examined the effects of a specific decentering strategy on individuals’ ability to resist chocolate. The strategy involved asking individuals to imagine themselves as a driver of a bus, driving toward their goals, and to view their thoughts as passengers on this bus. They compared the effects of this strategy with a mindfulness-based acceptance strategy and a relaxation (control) strategy. Participants in all three conditions were asked to use their strategy every time they felt like eating chocolate over a 5-day period. However, only those in the decentering group showed reduced chocolate consumption relative to those in the control condition. There was some evidence to indicate that this effect may have been brought about by reduced automaticity; scores on a habit index, taken before and after the 5-day period showed a significantly greater reduction in the decentering group compared to the control group and scores on this index after the 5-day period were also significantly correlated with chocolate consumption. However, no measures of goal accessibility were taken.

The current study extends this research by examining the effect of this decentering strategy on the cognitive accessibility of weight loss and healthy eating goals; goals that are typically incompatible with the consumption of chocolate. This was assessed using a word stem completion task in which participants are provided with the first three letters of a word and are asked to complete it with whatever word first comes to mind (Tiggemann et al., 2004). If the decentering strategy increases the cognitive accessibility of relevant goals, we would expect participants in the decentering condition to record more weight loss and healthy eating related words in the word stem completion task compared to those in the control condition. We would also expect the effect on weight loss related words to be larger among those who are dieting to lose weight and who indicate that weight management is important to them. Likewise we would expect the effect on healthy eating related words to be larger among those who indicate that healthy eating is important to them.

## Materials and Methods

### Participants

Participants were 90 university students (60 females, mean age = 20.75 years, *SD* = 2.71) who responded to email and poster advertisements to take part in a study on goal achievement. Since there were no previous studies employing the current version of the word stem completion task, a precise power analysis was not possible. However, the sample size of 45 participants per condition was informed by Tiggemann et al. (2004) and Jenkins and Tapper (2014). Participants received 30 course credits on completion of the study, which was approved by the City, University of London, Psychology Department Research Ethics Committee.

### Measures

#### Word Stem Completion Task

Participants were presented with a series of 20 word stems and asked to complete them to form the first word that came to mind. Each stem consisted of the first three letters of a word that could relate to weight loss/healthy eating or an alternative word unrelated to weight loss/healthy eating. For example ‘CAL…’ could be completed to form the word ‘CALORIE’ or ‘CALL.’ Fifteen stems could be completed to form weight loss related words and five stems could be completed to form healthy eating related words (see **Appendix A**). The word stems appeared individually on a computer screen and were presented in a new random order for each participant. The measure was scored by counting the number of healthy eating and weight loss related words recorded by each participant.

#### Diet and Motivation Questionnaire

Diet status was assessed by asking participants to indicate whether or not they were currently dieting to lose weight. Motivation for weight management and healthy eating were assessed by asking them to rate the extent to which they agreed with the statements ‘It is important to me to watch my weight’ and ‘It is important to me to try to eat a healthy diet’ respectively. Ratings were recorded on a five-point scale anchored by ‘strongly disagree’ and ‘strongly agree.’

### Decentering Manipulation

Participants allocated to the decentering condition were asked to imagine that they were the driver of a bus, driving toward their goals and that their thoughts were a bit like passengers on the bus; their job as the driver was to stick to their planned route, regardless of what their thoughts were saying. Participants were asked to practice this ‘thought technique’ for 5 min then to apply the technique for 3 min whilst looking at an image of an unwrapped chocolate bar.

In the control condition, participants were asked to imagine they were getting stressed about achieving their goals and that their muscles may start to tense or feel tired or uncomfortable; their job was to try to tense and relax their muscles regardless of how tired they felt. Participants were asked to practice this ‘relaxation technique’ for 5 min then to let their mind wander for 3 min whilst looking at an image of an unwrapped chocolate bar.

In both conditions all instructions were presented on a computer and participants were asked to press the space bar when they were ready to start practicing their given technique and applying it/letting their mind wander. A buzzer sounded at the end of the 5- and 3-min periods. The conditions were matched as far as possible, for example in terms of the chocolate image, text length and sentence structure, and use of the word ‘goals.’

### Procedure

Following collection of demographic details, E-prime was used to randomly allocate participants to the experimental and control conditions, present a brief written overview of the study and deliver the decentering/control task followed by the word stem completion task. The diet and motivation questionnaire was then administered using pen and paper.

## Results

Participants in the decentering and control conditions were well matched in terms of dieting status and motivation for weight management and healthy eating (see **Table 1**).

**Table 1:** Participant characteristics in the two conditions.

Characteristic	Decentering (*n* = 45)	Control (*n* = 45)
Percentage dieting to lose weight	27%	31%
Importance of watching weight, on a scale of 1–5 (mean, SD)	3.71 (1.01)	3.69 (1.15)
Importance of healthy eating, on a scale of 1–5 (mean, SD)	3.96 (1.09)	3.89 (1.05)

**Table 2** shows the number of weight and healthy eating related words generated by dieters and non-dieters in the decentering and control conditions. A 2 (condition) × 2 (diet status) × 2 (word type) ANOVA showed a main effect of condition on total number of goal-related words, *F*(1,86) = 17.99, *p* < 0.001, indicating that significantly more goal-related words were generated in the decentering group compared to the control group. However, there was no significant interaction between condition, diet status and word type, *F*(1,86) = 1.22, *p* = 0.27, thus the prediction that participants who were dieting to lose weight would generate more weight loss related words in the decentering condition compared to the control condition was not supported (though the means were in the predicted direction, see **Table 2**). Likewise, there was also no significant interaction between condition and diet status, *F*(1,86) = 2.02, *p* = 0.16, indicating that the overall increase in goal-related words occurred irrespective of whether or not participants were dieting to lose weight (though again, the means were in the predicted direction, see **Table 2**). Additionally, there was no main effect of diet status, *F*(1,86) = 0.69, *p* = 0.41, indicating that those who were dieting to lose weight did not generate more goal-related words overall.

**Table 2:** Number of weight loss and healthy eating related words generated by dieting and non-dieting participants in the decentering and control conditions.

Condition	Weight loss words, mean *(SD)*	Healthy eating words, mean *(SD)*	Total goal related words, mean *(SD)*
Decentering (*n* = 45)	4.53 (2.56)	1.29 (0.82)	5.82 (2.91)^a^
*Dieters (n = 12)*	*5.42 (2.50)*	*1.33 (1.16)*	*6.75 (2.77)*
*Non-dieters (n = 33)*	*4.21 (2.55)*	*1.27 (0.67)*	*5.48 (2.92)*
Control (*n* = 45)	2.60 (1.63)	1.20 (0.76)	3.80 (1.79)^a^
*Dieters (n = 14)*	*2.50 (1.40)*	*1.07 (0.48)*	*3.57 (1.45)*
*Non-dieters (n = 31)*	*2.65 (1.74)*	*1.26 (0.86)*	*3.90 (1.94)*

*^a^Means are significantly different, p < 0.001.*

Linear regression was used to test the effect of motivation for weight management on the relationship between condition and weight loss words generated. Condition was entered at time 1, motivation at time 2 and the interaction term between condition and motivation was entered at time 3. The dependent variable was the number of weight loss related words generated. As shown in **Table 3**, although the number of weight loss related words was significantly predicted by condition (*R*^2^ = 17%, *p* < 0.001), it was not significantly predicted by motivation for weight management (*R*^2^Δ = 0%, *p* = 0.92) or by the interaction between motivation and condition (*R*^2^Δ = 0%, *p* = 0.71), thus failing to support the prediction that the effect of condition on number of weight loss words would be stronger among those who were motivated to manage their weight.

**Table 3:** Linear regression model examining the main and moderating effect of motivation for weight management on number of weight loss related words generated (*n* = 90).

	Words generated		
	B	SE B	Beta
Step 1			
Constant	2.60	0.32	
Condition^a^	1.93	0.45	0.41^∗∗^
*R^2^*		0.17^∗∗^	
Step 2			
Constant	2.52	0.85	
Motivation	0.02	0.21	0.01
*R^2^*		0.17	
*Δ R^2^*		0.00	
Step 3			
Constant	2.25	1.10	
Condition^a^ × Motivation	0.16	0.43	0.14
*R*^2^		0.17	
*Δ R*^2^		0.00	

*^∗∗^p < 0.01. ^a^Control = 0, experimental = 1.*

Motivation for healthy eating showed a strong negative skew and was therefore dichotomised into low motivation (for ratings 1, 2, and 3) and high motivation (for ratings 4 and 5). As shown in **Table 4**, participants in the decentering condition with high motivation for healthy eating generated more healthy eating related words (1.34) compared to those with low motivation (1.15), and this pattern was reversed in the control condition (1.13 and 1.36 respectively). However, a 2 (condition) × 2 (motivation) ANOVA showed no significant interaction between condition and motivation, *F*(1,89) = 1.32, *p* = 0.25, thus failing to support the hypothesis that the effect of decentering on generation of healthy eating words would be stronger among those who were more motivated to eat healthily. There was also no main effect of condition, *F*(1,89) = 0.00, *p* = 0.98 and no main effect of motivation, *F*(1,89), *p* = 0.92.

**Table 4:** Number of healthy eating related words generated by participants with high and low motivation for healthy eating.

Condition	Healthy eating words, mean *(SD)*
Decentering (*n* = 45)	1.29 (0.82)
*Low motivation (n = 13)*	*1.15 (0.55)*
*High motivation (n = 32)*	*1.34 (0.90)*
Control (*n* = 45)	1.20 (0.76)
*Low motivation (n = 14)*	*1.36 (0.93)*
*High motivation (n = 31)*	*1.13 (0.67)*

## Discussion

The results showed that participants who engaged in a decentering strategy whilst viewing an image of a chocolate bar subsequently generated more weight loss and healthy eating related words in a word stem completion task, compared to those who engaged in mind wandering. These results suggest that the decentering strategy may increase the cognitive accessibility of constructs relating to weight loss and healthy eating. Since the activation of a valued goal tends to result in the inhibition of alternative goals (Shah et al., 2002), where participants are trying to lose weight or eat healthily this increased activation could help inhibit short term hedonic goals and in this way help individuals adhere to weight loss and healthy eating plans (Stroebe et al., 2013).

However, contrary to predictions, the results failed to show significant moderating effects of diet status, motivation for weight management and motivation for healthy eating. Nevertheless, the means were in the predicted directions. It is likely that the study was underpowered to detect a moderating effect of diet status given the relatively small numbers of dieters in the sample, and the fact that the sample size was calculated to detect a main effect rather than interaction effects (see section “Participants”). The data collected in this study should be useful for calculating effect sizes for future research. Similarly, the measures of motivation for weight management and for healthy eating may have reflected the use of a diverse set of strategies, such as exercising or regular weighing for weight management, and intake of a balanced diet or more fruit and vegetables for healthy eating. Thus these measures may have been only very weakly related to attempts to limit chocolate intake. Additionally, the word stem measure of healthy eating may have been less sensitive than the word stem measure of weight loss since it was based on just five words, as opposed to 15. This was in part due to difficulties in identifying appropriate healthy eating related words and stems. Future research may therefore benefit from targeting populations in which there are likely to be a higher proportion of dieters, restricting the focus of the study to dieting status and the accessibility of weight loss related goals, and using data from this study to ensure that the research is not underpowered.

An important question to consider is whether the decentering strategy employed in this study would be more effective than other strategies designed to increase the accessibility of weight loss or healthy eating goals, especially as this particular decentering strategy asked individuals to imagine they were ‘driving toward their goals.’ Arguably, simply telling individuals to think about their goals whenever they are tempted to eat chocolate could be just as effective (e.g., see van Koningsbruggen et al., 2011). In the present study the use of the term ‘goals’ was controlled for in both the practice phase and the application/mind wandering phase and the results suggested that the decentering strategy led to more goal activation than mind wandering. (In other words, when left to think about whatever they liked, participants did not spontaneously think about their goals more than they did when asked to use the decentering strategy.) However, future research would benefit from comparing the decentering strategy with a strategy that more explicitly prompts individuals to remember their goals. Alternatively, future research could examine other decentering strategies that do not refer to goals.

One reason the decentering strategy could be more effective than other strategies that simply prompt the individual to think about their goals, is that the decentering strategy may encourage individuals to notice the specific thoughts and feelings that precede particular behaviors. As mentioned previously, accurate identification of internal cues that elicit automatic responses may be a prerequisite for preventing those behaviors (Adriaanse et al., 2009), but individuals may find it difficult to accurately identify relevant cues without a period of introspection. In particular, it may be easier to prevent the initiation of an automatic response (by becoming aware of the cues that elicit it) rather than inhibit a response that has already been initiated (for example if the individual only notices that their behavior is inconsistent with their goals at the point at which they are already approaching the relevant object; Schachar et al., 2007; Jones et al., 2016). Becoming aware of cues that elicit behavior may also enhance self-control by allowing the individual to avoid cognitive elaboration, which may in turn prevent the development of desire (Kavanagh et al., 2005; May et al., 2012, 2015; see also Grabovac et al., 2011; Brewer et al., 2013). However, strategies that target decentering may not be necessary to bring about this type of increased awareness; instead mindfulness-based strategies based on increased present moment awareness of thoughts and feelings may be sufficient. Again, further research carefully controlling for the effects of such techniques would be helpful.

Finally, it is important to note that a key limitation of the present study is that the decentering strategy was applied in a context in which individuals were asked to view an image of a chocolate bar, rather than in a context in which they had the opportunity to consume chocolate. Thus the thoughts and feelings that the image elicited would not necessarily have been the same as those elicited prior to actually consuming chocolate. This could be addressed in future studies by using real food, available for participants to eat. This would have a further advantage of providing a measure of consumption that would in turn allow for tests of mediation. The study design could also be strengthened by including a baseline word stem completion task in order to more clearly establish that the decentering task increases goal accessibility. Additionally, collecting data on the body mass index of participants would be informative as this may influence their responses, and it would be important to establish whether the strategy is likely to be helpful among those who would benefit most.

## Conclusion

The results suggest that the decentering strategy employed in the present study may have increased the accessibility of weight loss and healthy eating related goals. It is possible that this increased accessibility was responsible for the effects on consumption found by Jenkins and Tapper (2014). This raises the question of whether the decentering strategy would be more effective than other strategies (for example using implementation intentions) that prompt the individual to think about their goals. One way in which the decentering strategy may offer an advantage is by helping individuals to accurately identify the specific thoughts and feelings that elicit behavior. However, this is likely to be achieved by all mindfulness-based present moment awareness strategies and may not be unique to decentering strategies. Further carefully controlled studies are needed to explore these different possibilities.

## Ethics Statement

This study was carried out in accordance with the recommendations of the British Psychological Society. The protocol was approved by the City, University of London Psychology Department Research Ethics Committee. All subjects gave written informed consent in accordance with the Declaration of Helsinki.

## Author Contributions

KT designed the study, contributed to data analysis, and prepared the manuscript. ZA contributed to the study design, programmed the study in E-prime, collected and analyzed the data, and contributed to the final manuscript.

## Conflict of Interest Statement

The authors declare that the research was conducted in the absence of any commercial or financial relationships that could be construed as a potential conflict of interest.

## Appendix A

### Target Weight Loss and Healthy Eating Related Words Used in the Word-Stem Completion Task

Weight loss related words: obese/obesity; calorie(s); binge; scale(s); diet; slim; slender; plump; skinny; cellulite; waist; thin; weight; restraint; willpower.

Healthy eating related words: sugar; saturate(s/d); balanced; health; mineral(s).

## References

[B1] Adriaanse M. A. de Ridder D. T. De Wit J. B. (2009). Finding the critical cue: implementation intentions to change one’s diet work best when tailored to personally relevant reasons for unhealthy eating. *Pers. Soc. Psychol. Bull.* 35 60–71. 10.1177/0146167208325612 19106078

[B2] Adriaanse M. A. de Ridder D. T. D. Evers C. (2011). Emotional eating: eating when emotional or emotional about eating? *Psychol. Health* 26 23–39. 10.1080/08870440903207627 20204980

[B3] Barsalou L. W. (2008). Grounded cognition. *Annu. Rev. Psychol.* 59 617–645. 10.1146/annurev.psych.59.103006.093639 17705682

[B4] Brewer J. A. Elwafi H. M. Davis J. H. (2013). Craving to quit: psychological models and neurobiological mechanisms of mindfulness training as treatment for addictions. *Psychol. Addict. Behav.* 27 366–379. 10.1037/a0028490 22642859 PMC3434285

[B5] Cleobury L. Tapper K. (2014). Reasons for eating ‘unhealthy’snacks in overweight and obese males and females. *J. Hum. Nutr. Diet.* 27 333–341. 10.1111/jhn.12169 24134077

[B6] Grabovac A. D. Lau M. A. Willett B. R. (2011). Mechanisms of mindfulness: a Buddhist psychological model. *Mindfulness* 2 154–166. 10.1007/s12671-011-0054-5

[B7] Jenkins K. T. Tapper K. (2014). Resisting chocolate temptation using a brief mindfulness strategy. *Br. J. Health Psychol.* 19 509–522. 10.1111/bjhp.12050 23678870

[B8] Jones A. Di Lemma L. C. Robinson E. Christiansen P. Nolan S. Tudur-Smith C. (2016). Inhibitory control training for appetitive behaviour change: a meta-analytic investigation of mechanisms of action and moderators of effectiveness. *Appetite* 97 16–28. 10.1016/j.appet.2015.11.013 26592707

[B9] Katterman S. N. Kleinman B. M. Hood M. M. Nackers L. M. Corsica J. A. (2014). Mindfulness meditation as an intervention for binge eating, emotional eating, and weight loss: a systematic review. *Eat. Behav.* 15 197–204. 10.1016/j.eatbeh.2014.01.005 24854804

[B10] Kavanagh D. J. Andrade J. May J. (2005). Imaginary relish and exquisite torture: the elaborated intrusion theory of desire. *Psychol. Rev.* 112 446–467. 10.1037/0033-295X.112.2.446 15783293

[B11] Lacille J. Ly J. Zacchia N. Bourkas S. Glaser E. Knauper B. (2014). The effects of three mindfulness skills on chocolate craving. *Appetite* 76 101–112. 10.1016/j.appet.2014.01.072 24503333

[B12] Mantzios M. Wilson J. C. (2015). Mindfulness, eating behaviours, and obesity: a review and reflection on current findings. *Curr. Obes. Rep.* 4 141–146. 10.1007/s13679-014-0131-x 26627097

[B13] May J. Andrade J. Kavanagh D. J. Hetherington M. (2012). Elaborated intrusion theory. A cognitive-emotional theory of food craving. *Curr. Obes. Rep.* 1 114–121. 10.1007/s13679-012-0010-2 15493072

[B14] May J. Kavanagh D. J. Andrade J. (2015). The elaborated intrusion theory of desire: a 10-year retrospective and implications for addiction treatments. *Addict. Behav.* 44 29–34. 10.1016/j.addbeh.2014.09.016 25306214

[B15] Moffitt R. Brinkworth G. Noakes M. Mohr P. (2012). A comparison of cognitive restructuring and cognitive defusion as strategies for resisting a craved food. *Psychol. Health* 27 74–90. 10.1080/08870446.2012.694436 22691136

[B16] National Institute for Health and Clinical Excellence (2014). *Obesity: Identification, Assessment and Management. Clinical Guideline.* Available at: https://www.nice.org.uk/guidance/cg189

[B17] Neal D. T. Wood W. Wu M. Kurlander D. (2011). The pull of the past: when do habits persist despite conflict with motives? *Pers. Soc. Psychol. Bull.* 37 1428–1437. 10.1177/0146167211419863 21859902

[B18] Olson K. L. Emery C. F. (2015). Mindfulness and weight loss: a systematic review. *Psychosom. Med.* 77 59–67. 10.1097/PSY.0000000000000127 25490697

[B19] O’Reilly G. A. Cook L. Spruijt-Metz D. Black D. S. (2014). Mindfulness-based interventions for obesity-related eating behaviours: a literature review. *Obes. Rev.* 15 453–461. 10.1111/obr.12156 24636206 PMC4046117

[B20] Papies E. K. Pronk T. M. Keesman M. Barsalou L. W. (2015). The benefits of simply observing: mindful attention modulates the link between motivation and behaviour. *J. Pers. Soc. Psychol.* 108 148–170. 10.1037/a0038032 25347126

[B21] Schachar R. Logan G. D. Robaey P. Chen S. Ickowicz A. Barr C. (2007). Restraint and cancellation: multiple inhibition deficits in attention deficit hyperactivity disorder. *J. Abnorm. Child Psychol.* 35 229–238. 10.1007/s10802-006-9075-2 17351752

[B22] Schumacher S. Kemps E. Tiggemann M. (2017). Acceptance-and imagery-based strategies can reduce chocolate cravings: a test of the elaborated-intrusion theory of desire. *Appetite* 113 63–70. 10.1016/j.appet.2017.02.012 28196711

[B23] Shah J. Y. Friedman R. Kruglanski A. W. (2002). Forgetting all else: on the antecedents and consequences of goal shielding. *J. Pers. Soc. Psychol.* 83 1261–1280. 10.1037/0022-3514.83.6.1261 12500810

[B24] Stroebe W. Van Koningsbruggen G. M. Papies E. K. Aarts H. (2013). Why most dieters fail but some succeed: a goal conflict model of eating behavior. *Psychol. Rev.* 120 110–138. 10.1037/a0030849 23230892

[B25] Tapper K. (2017). Can mindfulness influence weight management related eating behaviors? If so, how? *Clin. Psychol. Rev.* 53 122–134. 10.1016/j.cpr.2017.03.003 28347881

[B26] Tapper K. (2018). Mindfulness and craving: effects and mechanisms. *Clin. Psychol. Rev.* 59 101–117. 10.1016/j.cpr.2017.11.003 29169665

[B27] Tiggemann M. Hargreaves D. Polivy J. McFarlane T. (2004). A word-stem completion task to assess implicit processing of appearance-related information. *J. Psychosom. Res.* 57 73–78. 10.1016/S0022-3999(03)00565-8 15256298

[B28] van Koningsbruggen G. M. Stroebe W. Papies E. K. Aarts H. (2011). Implementation intentions as goal primes: boosting self-control in tempting environments. *Eur. J. Soc. Psychol.* 41 551–557. 10.1002/ejsp.799

[B29] Verhoeven A. A. Adriaanse M. A. Evers C. de Ridder D. T. (2012). The power of habits: unhealthy snacking behaviour is primarily predicted by habit strength. *Br. J. Health Psychol.* 17 758–770. 10.1111/j.2044-8287.2012.02070 22385098

